# A systematic review of major evaluation metrics for simulator-based automatic assessment of driving after stroke

**DOI:** 10.1016/j.heliyon.2024.e32930

**Published:** 2024-06-17

**Authors:** Pittawat Taveekitworachai, Gunt Chanmas, Pujana Paliyawan, Ramita Thawonmas, Chakarida Nukoolkit, Piyapat Dajpratham, Ruck Thawonmas

**Affiliations:** a Graduate School of Information Science and Engineering, Ritsumeikan University, 2-150 Iwakura-cho, Ibaraki, 567-8570, Osaka, Japan; b Ritsumeikan Center for Game Studies, Ritsumeikan University, 56-1 Toji-in Kitamachi, Kita, 603-8577, Kyoto, Japan; c School of Tropical Medicine and Global Health, Nagasaki University, 1-12-4 Sakamoto, Nagasaki City, 852-8523, Nagasaki, Japan; d School of Information Technology, King Mongkut's University of Technology Thonburi, 126 Pracha Uthit Road, Bang Mod, Thung Khru, 10140, Bangkok, Thailand; e Department of Rehabilitation Medicine, Faculty of Medicine Siriraj Hospital, Mahidol University, 2 Wanglang Road, Siriraj, Bangkok Noi, 10700, Bangkok, Thailand; f Department of Information Science and Engineering, College School of Information Science and Engineering, Ritsumeikan University, 2-150 Iwakura-cho, Ibaraki, 567-8570, Osaka, Japan

**Keywords:** Stroke, Driving assessment, Driving simulator, Driving performance, Systematic review

## Abstract

**Background:** Simulator-based driving assessments (SA) have recently been used and studied for various purposes, particularly for post-stroke patients. Automating such assessment has potential benefits especially on reducing financial cost and time. Nevertheless, there currently exists no clear guideline on assessment techniques and metrics available for SA for post-stroke patients. Therefore, this systematic review is conducted to explore such techniques and establish guidelines for evaluation metrics.

**Objective:** This review aims to find: (a) major evaluation metrics for automatic SA in post-stroke patients and (b) assessment inputs and techniques for such evaluation metrics.

**Methods:** The study follows the PRISMA guideline. Systematic searches were performed on PubMed, Web of Science, ScienceDirect, ACM Digital Library, and IEEE Xplore Digital Library for articles published from January 1, 2010, to December 31, 2023. This review targeted journal articles written in English about automatic performance assessment of simulator-based driving by post-stroke patients. A narrative synthesis was provided for the included studies.

**Results:** The review included six articles with a total of 239 participants. Across all of the included studies, we discovered 49 distinct assessment inputs. Threshold-based, machine-learning-based, and driving simulator calculation approaches are three primary types of assessment techniques and evaluation metrics identified in the review.

**Discussion:** Most studies incorporated more than one type of input, indicating the importance of a comprehensive evaluation of driving abilities. Threshold-based techniques and metrics were the most commonly used in all studies, likely due to their simplicity. An existing relevant review also highlighted the limited number of studies in this area, underscoring the need for further research to establish the validity and effectiveness of simulator-based automatic assessment of driving (SAAD).

**Conclusions:** More studies should be conducted on various aspects of SAAD to explore and validate this type of assessment.

## Highlights

• Systematic review of major metrics in simulator-based assessment of driving.
• Six articles were included and classified into threshold- and ML-based metrics.
• Threshold-based metrics have the highest uses in included studies.
• Future research should focus on various components of driving assessment system.

## 1 Introduction

Driving is an essential skill for personal and professional life. It not only provides freedom and independence [1] for leisure activities such as road trips and grocery shopping but is also a requirement for many jobs, with over a 30% of positions in US in 2016 requiring driving [2]. For post-stroke patients, who may experience cognitive impairments [3], driving can be particularly important. Driving allows them to lead an independent life and engage in out-of-home activities [4]. In fact, research indicates that stroke survivors who are able to drive experience a higher quality of life [5].

Driving is a multifaceted task that necessitates the possession of numerous skills to perform it safely [6]. As a result, driving tests are usually required to assess an individual's ability to drive. Many countries have a standard driving license examination [7] that evaluates various aspects of an individual's driving skills to ensure their competence in operating a vehicle safely. However, post-stroke patients typically experience a decline in their cognitive abilities, which are crucial for safe driving [8]. Therefore, there is a need for reassessment of their driving abilities after a stroke. Driving assessment, which is a tool used to evaluate an individual's driving ability, is essential for determining their capability to drive.

There are three main types of driving assessment used to evaluate an individual's ability to drive [9]. The first type is the cognitive driving assessment (CA), which is used to preliminarily assess fundamental cognitive abilities related to driving before proceeding with further evaluation processes. Examples of CA tools include the Useful Field of View Test, the Trail Making Test, and the Key Search Test for testing visual processing speed, attention and executive functioning, and visual search skills, respectively [10]. The second type is on-road driving assessment (OA), which is considered the gold standard [11] because it requires the test-taker to drive in an actual vehicle and be evaluated by a human assessor. However, on-road driving assessment has a few key disadvantages, such as safety concerns for road users, high cost, and a limited variety of route scenarios [12]. To mitigate these issues, simulator-based assessment (SA) was introduced. SA imitates the on-road driving experience using a driving simulator while overcoming the disadvantages of on-road assessment.

SA has been adopted for various use cases for post-stroke patients, such as predicting actual driving abilities [13], as part of driving rehabilitation programs [14], and as a pre-screening tool [15], due to its numerous benefits. SA has several advantages over OA, including more affordable to test takers [16]. However, the validity and reliability of SA remain subjects of ongoing investigation [17].

SA's versatility allows for the simulation of diverse driving scenarios, conditions, and performance metrics, facilitating both customized rehabilitation programs and controlled research environments [6], [18]. SA comes with its unique benefits, allowing the driver to experience various types of driving scenarios in safe conditions without the risks of real-life danger [19].

By simulating various driving scenarios, SA provides a comprehensive assessment of driving performance, as some of the scenarios can be designed similar to driving license tests. The assessment assesses various capabilities of test takers, some found in driving license tests, including visual attention [20]; reaction time [13], [21]; and decision-making based on traffic rules (e.g., [13], [15], [21]), and various driving scenarios such as urban driving, highway navigation, and rural cruising [6]. Moreover, SA generates performance metrics comparable to those of OA, including signal uses [22], [23], speed (e.g., [13], [15], [21]), and distance between vehicles [15], [22], [23]. This comprehensive assessment capability positions SA as a valuable tool for evaluating driving proficiency.

At present, SA also has associated disadvantages, which are also present in OA, such as the requirement for certified personnel known as Certified Driving Rehabilitation Specialists (CDRS) to assist post-stroke patients who need further assistance or evaluation. However, the number of CDRS is inadequate [11], [24] as only 370 CDRS exist in the US and Canada. The shortage of CDRS poses a challenge to the assessment process. Automating the evaluation process of SA could potentially help reduce the burden on CDRS and mitigate this issue.

Nonetheless, there are currently no guidelines or comprehensive lists of metrics and techniques used for simulator-based automatic assessment of driving (SAAD) for post-stroke patients (SAAD-PS). This lack of standardization could make it challenging to automate the process. This review aims to alleviate this issue by providing a comprehensive overview of the current assessment techniques and metrics presented in the literature on SAAD-PS.

A similar review conducted by Hird et al. [9] provided a comprehensive overview of driving assessment and categorized their findings into three types, CA, OA, and SA, as previously described, with a notable observation that there was a limited amount of studies utilizing SA compared to other types of assessment (three out of 22 of their included studies). Therefore, to better understand the current situation in this area, we aim to conduct a systematic review focusing on automated SA. With advancements in technology, the adoption of SA is likely to increase for various scenarios, and our review could potentially address the issues of the lack of guidelines and assess the current state of the field while also reviewing the latest techniques used, which incorporate the improved technologies. Specifically, our review focuses on assessment techniques, evaluation metrics, and associated inputs used in recent literature. Compiling a list of current techniques should lead to the development of better SAAD-PS.

The main objectives of this review are as follows:

1. To find major evaluation metrics for SAAD-PS.
2. To find assessment inputs and techniques associated with such evaluation metrics.

## 2 Materials and methods

In this review, the PRISMA 2020 guideline [25] was applied. The fourth author, RaT, verified the review process. The registered protocol is available on OSF with the following DOI https://doi.org/10.17605/OSF.IO/DHU7W. We searched PubMed, Web of Science, ScienceDirect, ACM Digital Library, and IEEE Xplore Digital Library. We conducted our initial search on January 27, 2022, followed by four updated searches on June 21, 2022, November 11, 2022, February 22, 2023, and March 9, 2024.

### 2.1 Search strategy development process

We used PICO [26] to define the scope of relevant studies. The patient (P) criterion focuses on post-stroke patients. The intervention (I) criterion is defined as SAAD. The control (C) criterion involves different inputs, techniques, and evaluation metrics used in each study. The outcome (O) criterion is related to analyses of the automatic performance assessment.

Then we constructed inclusion and exclusion criteria based on PICO. From criteria items, we identified search terms, a search query template, and associated search parameters. Finally, we developed a set of search terms and search parameters for each database. Table S1 in the Supplementary Material lists all of the search parameters and queries for each database.

### 2.2 Eligibility criteria

#### 2.2.1 Inclusion criteria

1. Post-stroke survivors with driving experience
2. Studies including techniques for SAAD
3. Studies written in English

#### 2.2.2 Exclusion criteria

1. Studies involving only adolescents
2. Studies involving only manual driving assessment
3. Studies involving only preliminary cognitive evaluation for driving
4. Studies involving only on-road driving assessment
5. Studies involving only driver monitoring
6. Studies involving only automated driving

### 2.3 Search terms

Based on the inclusion and exclusion criteria, we constructed a search query template as follows:

(stroke OR cerebrovascular OR “cerebral vascular”) AND (driving OR drive) AND (test OR assess* OR measur* OR evaluat*) AND simulat*

The asterisk symbol (*) indicates a wildcard; for example, simulat* is equivalent to *simulate*, *simulation*, *simulator*, etc. The quotation mark (*“...”*) specifies an exact word search, i.e., without variation. The parenthesis symbol (*(...)*) groups the search terms that belong to the same logical group. The *AND* and *OR* symbols are logical AND and logical OR, respectively.

### 2.4 Search parameters

The initial search results include articles published from January 1, 2010, to December 31, 2021. The first, second, third, and fourth updated search results include articles published from January 1, 2022, to June 21, 2022; from June 22, 2022, to October 31, 2022; from November 1, 2022, to December 31, 2022; and from January 1, 2023, to December 31, 2023, respectively. Only journal articles are included in this review.

### 2.5 Selection process

The first and second authors, PT and GC, independently assessed the eligibility of studies using the inclusion and exclusion criteria. The screening process used Rayyan^1^ as a platform for article de-duplication and blind assessment. All identified articles were assessed with their title then with their abstract. Later, the full-text version of each qualified article was retrieved and evaluated for inclusion in the review. Any disagreements about study eligibility in any stages were resolved through discussion with the third author, PP. Considering all searches, the inter-rater reliability between PT and GC is 99.57%.

### 2.6 Data extraction process

Data for analysis and synthesis were extracted from each included study using a template which is available in Supplementary Material Table S2. The extracted data for each study consist of the (a) input data used by the driving assessment technique, (b) driving simulator tool, (c) assessment technique, (d) evaluation metric, and (e) demographic of participants. PT extracted the data, and GC independently checked for their accuracy. Disagreements in the extracted data were discussed and resolved with other authors, PP, CN, and RuT.

### 2.7 Data synthesis process

PT did the narrative synthesis. We chose narrative synthesis because we were unable to conduct a meta-analysis due to the heterogeneity in how descriptions are interpreted, e.g., not all included studies gave a clear definition for collisions, making us unsure whether collisions count when hitting only another automobile or not. The information listed below was considered for organizing extracted data into categories.

1. **Objectives**: main objectives of each study.
2. **Simulator tools**: driving simulators used in a driving performance assessment process.
3. **Participant demographics**: the number of post-stroke and healthy participants, e.g., 80 post-stroke patients and 60 healthy controls.
4. **Assessment inputs**: data required by assessment techniques, e.g., speed, total runtime, and number of throttle use. Inputs are categorized into one of five categories: error, vehicle property, count, speed and acceleration, and time.
5. **Driving scenarios**: driving scenarios configured on the driving simulator were utilized by each study to collect assessment inputs.
6. **Assessment techniques**: data transformation processes. They turn assessment inputs into values used by evaluation metrics, e.g., summation of errors, machine learning (ML) models, and driving simulator calculation.
7. **Evaluation metrics**: rules for classifying information from assessment techniques into a final category, e.g., people with a score over 80 are PASS. Both techniques and metrics belong to one of three categories: threshold-based, ML-based, and driving simulator calculation approaches.
8. **Results**: results of participants were assessed according to the evaluation metrics from each study. In cases where the study focused on developing ML models, the performance metrics of the model are extracted.

An assessment-input-overview table, Table S3 in the Supplementary Material, was constructed to provide an overview of the results and enhance the narrative.

The results from the included studies were organized and summarized in four tables (Table 1, Table 2, Table 3, Table 4). We did not conduct a risk of bias assessment for each article included because the data of interest, being categorical text, had a certain level of ambiguity. Moreover, as almost all the studies pointed towards the same effect direction, we did not investigate potential sources of heterogeneity among the study results further. Canonical sensitivity analysis and certainty assessment, typically required for intervention studies, were deemed not applicable to our work.

**Table 1:** General characteristics of the included studies where **IS**, **HS** and **SAH** represent ischemic stroke, hemorrhagic stroke, and subarachnoid hemorrhage, while **R**, **L**, and **B** represent left, right, and bilateral lesions, respectively.

Study	Objectives	Simulator tools	Participant demographic
McKay et al. [22], [22]	Compared post-stroke patients' self-awareness of neuropsychological and driving simulator performances with those of healthy controls.	Doron AMOS-II	Post-stroke patients: 30 Age: 54.3 ± 9.1 R: 9/L: 17/B: 4 Healthy controls: 30 Age: 48.5 ± 13

Akinwuntan et al. [13]	Assessed the predictability of the proposed US version of stroke driver screening assessment.	STISIM Drive® Model 400	Post-stroke patients: 15 Age: 52 ± 12 IS: 10/HS: 5, R: 7/L: 7/B: 1 Healthy controls: 16 Age: 40 ± 16

Motta et al. [23], [23]	Examined the connection between post-stroke executive function and driving ability.	STISIM Drive®	Post-stroke patients: 19 Age: 70.1 ± 8.9 Healthy controls: 22 Age: 64.8 ± 6.7

Park [21], [21]	Examined the connection between R or L post- stroke patients and errors during driving.	GDS-300® developed by Gridspace	Post-stroke patients: 30 Age: 47 ± 12.96 R: 16/L: 14/B: 0

Hird et al. [27], [27]	Examined differences in driving performance between IS and SAH post-stroke patients.	STISIM Drive®	Post-stroke patients: 30 Age: 59.2 ± 11.5 IS: 15 (R: 8/L: 6/B: 1) SAH: 15 Healthy controls: 20 Age: 62 ± 10.6

Jeon et al. [15]	Developed a driving performance assessment system for post-stroke drivers utilizing AutoML for pre-screening and providing comprehensive details for rehabilitation purposes.	STISIM Drive®	Post-stroke patients: 18 Healthy controls: 9

**Table 2:** Assessment inputs and driving scenarios utilized by each included study, where ^⋆^ denotes inputs that are **not** directly recorded by the simulator.

Study	Assessment inputs	Driving scenarios
McKay et al. [22], [22]	**Table:** 1. Speed 2. Stop distance 3. Lane placement 4. Traffic signal use 5. Hazard avoidance 6. Obeying traffic signs and signals	**Table:** Number of driving scenarios: 4 Driving scenarios: Areas: residential, business, and rural 1. Residential and light business traffic 2. Rural traffic and roadways (including lane changes) 3. Challenging situations that require forethought and quick response time (e.g., near collisions, emergency vehicles) 4. A skills track module that includes assessment of brake reaction, front-end parking, and distance estimation.

Akinwuntan et al. [13]	**Table:** First course: None (practice course) Second course: 1. Time to collision 2. Number of edge crossings 3. Number of centerline crossings time 4. Speed limit violations 5. Collisions 6. Number of pedestrians hit 7. Total runtime Third course: 1. Simple reaction time 2. Complex reaction	**Table:** Number of practice sessions: 1 Practice session details: 2.4 km course with very light traffic. Drive as in real life while obeying all traffic rules Number of driving scenarios: 2 Second course: 15.3 km course with moderate traffic density Areas: rural, 2-lane roads; urban, 4-lane roads; and highway, 6-lane roads Speed limits: 48.3-112.6 km/h Lane markings: standard Intersections: with and without traffic signals Pedestrians, traffic lights, and required vehicular interactions Third course: 2.4 km course, 2-lane road Speed limit: 72 km/h STOP signs: 5

Motta et al. [23], [23]	**Table:** 1. Number of collisions, excluding pedestrians 2. Number of collisions with pedestrians 3. Driver stops at an appropriate distance from traffic lights, stop signs and obstacles^⋆^ 4. Driver did not overtake when unsafe, allowed adequate room, and stopped at yellow traffic lights^⋆^ 5. Kilometers over speed limit^⋆^ 6. Number of center line crossings 7. Number of road edge excursions 8. Driver appropriately used indicators to give warning and future diverging movements^⋆^	**Table:** Number of practice sessions: 1 Duration of practice session: 5 minutes Number of driving scenarios: 4 Areas: city and suburban Driving scenarios: 1. Basic intersections 2. Complex intersections 3. Curved roads 4. Emergency braking

Park [21], [21]	**Table:** 1. Total driving errors - Failed to use seat belt - Exceeded speed limit - Turn signal errors - Drop out the course - Crossed center line - Accidents - Brake reaction time (sec)	**Table:** Number of practice sessions: 1 Practice session details: Exercise mode of the simulator Number of driving scenarios: 1 Areas: downtown Seoul, South Korea, and highway Driving conditions: various buildings, moving cars, traffic signals, and road signs

Hird et al. [27], [27]	**Table:** 1. Total driving - Collision - Centerline crossings - Road edge excursions - Stop signs - Speed exceedances	**Table:** Number of driving scenarios: 1 Area: city Driving conditions: 1. Straight driving 2. Right turn 3. Left turn 4. Left turn with oncoming traffic
Jeon et al. [15]	**Table:** 1. Driver's longitudinal acceleration 2. Driver's lateral acceleration 3. Driver's longitudinal velocity (ft/s) 4. Driver's lateral velocity 5. Driver's lateral lane position with respect to the roadway dividing line 6. Vehicle curvature 7. Current roadway curvature 8. Vehicle heading angle 9. Steering wheel angle input 10. Longitudinal acceleration due to the throttle 11. Longitudinal acceleration due to the brakes 12. Running compilation of the crashes that the driver has been involved in 13. Driver's longitudinal velocity 14. Steering input counts 15. Throttle input counts 16. Braking input counts 17. Steering wheel rate 18. Minimum time to collision between the driver and all vehicles in the driver's direction 19. Minimum range between the driver and all vehicles in the driver's 20. Minimum time to collision between the driver and all vehicles opposing the driver's direction 21. Minimum range between the driver and all vehicles opposing the driver's direction	**Table:** Number of driving scenarios: 13 Driving scenarios: Area: urban, 1-way straight road 1. Traffic light (green), speed limit sign (70 km/h) 2. Traffic light (green), speed limit sign (70 km/h) 3. Traffic light (red), speed limit sign (70 km/h) 4. A parked car suddenly enters and exits ego vehicle's lane. 5. A parked car comes into driver's lane, slowly goes, then leaves. Area: highway, 2-way straight road 6. Speed limit sign (100 km/h) 7. Speed limit sign (100 km/h) 8. Speed limit sign (100 km/h) Area: rural. 1-way curvy road 9. Left curve sign, speed limit sign (100 km/h) 10. Right curve sign, speed limit sign (100 km/h) 11. Left curve sign, speed limit sign (100 km/h) 12. Right curve sign, speed limit sign (100 km/h) 13. Oncoming car is heading into driver's lane.

**Table 3:** Driving assessment details of each included study comprising techniques, criteria, and their results. In the ‘Results’ column, driving scores and accuracy are reported as mean ± standard deviation (SD), while pass rates are reported in percentages. **IS** and **SAH** represent ischemic stroke and subarachnoid hemorrhage, while **R** and **L** represent left and right lesions, respectively.

Study	Assessment techniques	Evaluation criteria	Results
McKay et al. [22], [22]	**Table:** Score calculation is given by the simulator.	**Table:** Not mentioned	**Table:** Performance comparison: Post-stroke patients: 38.0 ± 12.5 Healthy controls: 50.0 ± 10.0 Observation: The stroke group performed significantly worse than the control group on the driving simulator evaluation (*p* < .001, *η*^2^ = .22).

Akinwuntan et al. [13]	**Table:** An established algorithm derived from age-based normative values of all the inputs.	**Table:** Driving performance rating: 1. PASS if score is better or within the normative values. 2. FAIL if score if below the normative values.	**Table:** Pass rates: Post-stroke patients: 46.67% Healthy controls: 93.75% Observation: This study focused on developing a screening assessment battery and utilized performances from the simulator as ground truths.

Motta et al. [23], [23]	**Table:** Driving score calculation based on six assessment criteria according to the modified version of 1. West Australian licensing standards 2. Previous studies on the Curtin University driving simulator.	**Table:** Not mentioned	**Table:** Performance comparison: Post-stroke patients: 5.5 ± 3.0 Healthy controls: 3.2 ± 1.7 Observation: The control group performed better than the stroke participants with statistical significance (*p* = 0.005).

Park (2015)	**Table:** The maximum score of 100 is deducted by the total error score tracked by the simulator.	**Table:** FAIL: score is less than 80	**Table:** Performance comparison: R: 32.12 ± 18.47 L: 18.64 ± 16.57 Observation: There were significant differences in total driving error scores between R and L patients (*p* < 0.05).

Hird et al. [27], [27]	**Table:** Sum of driving errors.	**Table:** FAIL: Number of total driving errors ≥ 3 SDs above the mean number of errors committed by control drivers.	**Table:** Performance comparison and pass rates: Post-stroke patients: 21.2 ± 13.9 (30%) Healthy controls: 10.9 ± 5.7 (0%) Observation: Nine patients (IS: 4, SAH: 5) exhibited driving impairment, committing more than 3 SDs above the mean of errors committed by healthy controls (>28 total driving errors).

Jeon et al. [15]	**Table:** Eleven classifiers, built with AutoML feature of MATLAB®, using deep features from five pre-trained models with a resampling method.	**Table:** Driving suitability decided the output (PASS or FAIL) using an assessment classifier.	**Table:** Classifier performance: F1-score: 0.88 ± 0.08 Accuracy: 0.89 ± 0.07 Recall: 0.88 ± 0.08 Precision: 0.90 ± 0.07 Observation: This study focused on developing high-accuracy classifiers using data collected by the simulator as features (inputs).

**Table 4:** Assessment inputs used in the study.

Study	Total	Error	Vehicle	Count	Speed	Time
McKay et al. [22], [22]	6	0	2	3	1	0
Akinwuntan et al. [13]	9	5	0	0	0	4
Motta et al. [23], [23]	8	5	0	3	0	0
Park [21], [21]	7	6	0	0	0	1
Hird et al. [27], [27]	5	5	0	0	0	0
Jeon et al. [15]	21	1	8	3	7	2

## 3 Results

### 3.1 Search results

**Figure 1:**
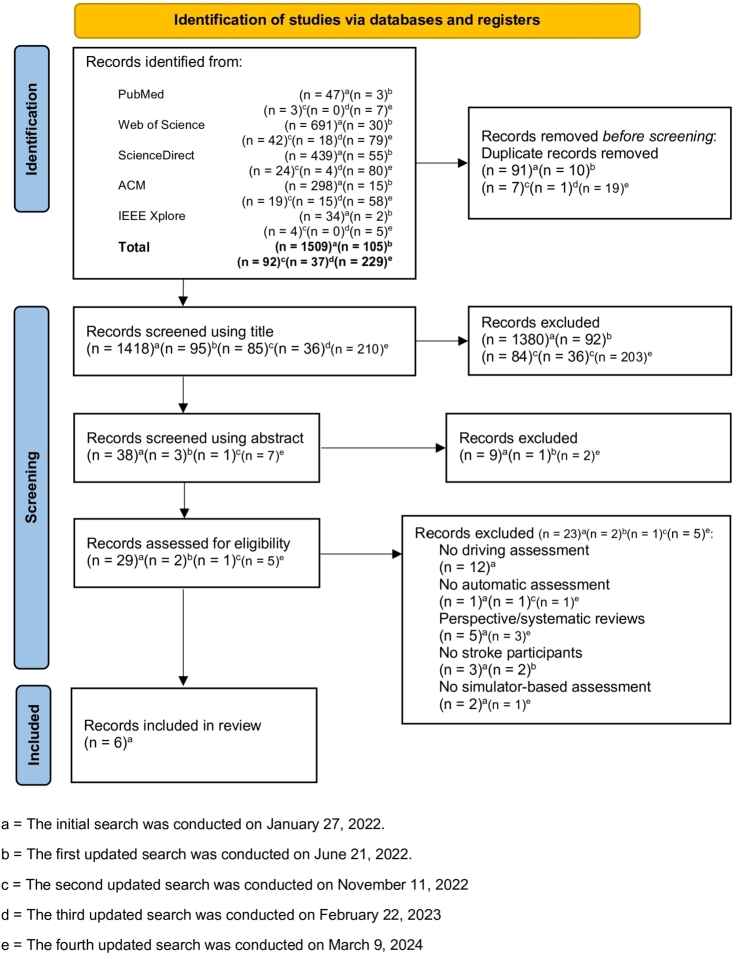
PRISMA flow diagram indicating results of the identification and screening process for included and excluded articles.

Initial search results contained 1,509 articles across five databases. After de-duplication of them, 1,418 articles remained for screening with the title. There were 38 remaining articles for the abstract screening after the title screening. Only 29 articles remained for full-text screening. Eventually, six articles were included in this review after the full-text screening. The unqualified articles were filtered out due to reasons listed in Fig. 1 as a part of the PRISMA flow diagram.

The first updated search was performed which yielded 105 additional articles. There were 95 articles remaining after duplicate removal. Almost all articles were excluded after screening using their titles; only three articles were left for abstract screening. Only two articles remained for screening with full text. However, none of them were included in the review because they did not involve only stroke participants.

The second updated search resulted in 92 articles, from which 85 articles remained after automatic de-duplication. Only one article passed the title and abstract screenings, but it was excluded after the full-text screening because it did not involve automatic assessment of driving. After conducting the third updated search, 37 articles were retrieved, but 36 remained after using an automatic de-duplication process. However, none of these articles passed the initial title screening, so no further screening was conducted.

The fourth updated search resulted in 229 articles, which reduced to 210 articles after de-duplication. Following the title screening, seven articles remained for abstract screening. Two articles were excluded during the abstract screening as they were unrelated to driving assessment. The remaining five articles underwent full-text screening, resulting in three exclusions due to being a systematic review. The two remaining articles were excluded because one did not involve SA, and the other lacked automatic assessment.

In summary, we found six articles satisfied our criteria to be included in this review. Of six articles, all, except Motta et al. [23], [23], were published in four unique journals with an impact factor. The mean and standard deviation of impact factor of these four journals are 3.4 ± 0.7. Including the publishing journal of Motta et al. [23], [23], the mean and standard deviation of h5-index of these five journals are 87.6 ± 76.4.

### 3.2 General characteristics of included studies

There was heterogeneity across the included studies. Two studies [21], [27] focused on driving ability comparison between groups, e.g., between post-stroke patients and healthy controls. Three studies [13], [22], [23] centered around finding associations between driving assessment scores and other metrics. One study [15] dedicated itself to the development of a driving performance assessment system.

Park [21], [21] and Hird et al. [27], [27] aimed to compare driving performances between post-stroke patients with right (R) and left (L) lesions, and between ischemic stroke (IS) and subarachnoid hemorrhage (SAH) types, respectively. Park [21], [21] found a statistically significant difference in driving errors committed by post-stroke patients with R and L; specifically, individuals with R exhibited a higher total number of driving errors compared to those with L. Hird et al. [27], [27] observed differences in specific aspects of driving performance between IS and SAH post-stroke patients. On the other hand, McKay et al. [22], [22], Akinwuntan et al. [13], and Motta et al. [23], [23] investigated driving performances in relation to self-awareness of abilities, the predictability of their proposed US version for stroke driver screening assessment, and the connection between executive functions and driving performances.

McKay et al. [22], [22] found that not only did post-stroke drivers perform worse (with statistical significance) compared to healthy controls, but these drivers also overestimated their capabilities in driving. Akinwuntan et al. [13] found high predictability of the US version performance for both post-stroke patients (87%) and healthy controls (88%). However, the study by Akinwuntan et al. [13] is preliminary, and further studies are required to establish greater predictability. Motta et al. [23], [23] found that not only did post-stroke patients perform worse than healthy controls in all cognitive and driving simulator tests, but there were also associations between some specific cognitive tests and driving scores. Jeon et al. [15] achieved high-accuracy classifiers for predicting various aspects of driving performance, including driving suitability (81%-99%).

Furthermore, we found that post-stroke patients performed worse than healthy controls in all cases for studies involving comparisons between post-stroke patients and healthy controls [13], [22], [23]. Two studies [22], [23] reported that their results were statistically significant, and one study [13] found that the pass rate of post-stroke patients is less than half compared to over 90% of healthy controls. This observation aligns with the general understanding that driving ability after stroke is degraded due to several impairments and skills required for driving [28]. Details of the included articles are shown in Table 1, Table 2, Table 3.

### 3.3 Participant demographics

There are a total of 239 participants composed of 142 post-stroke patients and 97 healthy controls across the six included studies. The average number of participants in each study is 39.8, 23.7 for post-stroke patients and 16.2 for healthy controls. Participants of one study [21], [21] only consisted of post-stroke patients without healthy controls, while all the other studies had both groups. Average age of participants in the included studies is in range of 40 to 70.1 years old.

### 3.4 Driving scenarios

Across six included studies, 25 driving scenarios were identified, where Jeon et al. [15] utilized the highest number of driving scenarios at 13 scenarios while the rest of the studies utilized one to four driving scenarios. The high number of driving scenarios utilized by Jeon et al. [15] is potentially attributed to the fact that this study investigated the development of SAAD-PS system instead of finding the actual performance of the participants. We found another surprising result of lacking (or lack of report) for practice sessions. Only three studies [13], [21], [23] reported the utilization of practice sessions for participants to familiarize with the systems. Practice sessions lasted for a few minutes and did not utilize the scenarios used for collecting assessment inputs. All included studies except Hird et al. [27], [27] utilized various types of areas, e.g., urban, highway, and rural, to comprehensively assess the performance of the participants. Therefore, the limited use of driving scenarios restricted the interpretation of the study by Hird et al. [27], [27].

### 3.5 Assessment inputs

Assessment inputs are data gathered and fed into a given assessment technique. There are many inputs that the designers of an assessment technique can choose to collect. We identified 49 unique assessment inputs across the studies.

Table 4 shows a breakdown of assessment inputs in use. In this table, **Total** denotes the total number of assessment inputs used in each study, while **Error**, **Vehicle**, **Count**, **Speed**, and **Time** denote the number of assessment inputs belonging to the error, vehicle property, count, speed and acceleration, and time categories, respectively. The mean of inputs per study is 9.33, and the standard deviation is 5.89. The exhaustive list of all inputs used in each study is shown in Supplementary Material Table S3.

An error input measures the degree that the driver violates a rule or condition defined by the researchers or the software program in use, e.g., collisions, pedestrian hits, and centerline crossings. Four of the included studies [13], [21], [23], [27] used at least five unique errors. This likely indicates that errors are good indicators of driving performance.

Inputs in the vehicle property category are metrics gathered from vehicles, e.g., the vehicle's headway angle, stopping distance, and wheel angle. A count input shows the number of occurrences of a specific event, for instance, the number of traffic signal uses, the number of break uses, and the number of throttle uses. The speed and acceleration category is a subset of the vehicle property category. A measure of time for any actions/observations of interest falls into the time category, e.g., total runtime, time to collision, and simple reaction time.

### 3.6 Simulator tools

A driving simulator is the central part in this line of research. The simulator not only provides the user-facing interface for experiments but also defines the kinds of experiments that could be performed. Driving simulators by STISIM Drive® and Gridspace are commercial products that come with both hardware and software components designed for various purposes and can be installed with minimal configuration. In contrast, Doron's Advanced Mobile Operations Simulator (AMOS) II is specifically built for training purposes.

STISIM Drive® provides many models of a driving simulator. It is the most popular choice of simulators in the included studies [13], [15], [23], [27] with an ability to design custom scenarios with a range of metrics for further analysis and classification. Gridspace developed the GDS-300 simulator. GDS-300 comes with five tests: reaction time, judgment, speed anticipation, steering wheel-pedal operation, and on-road driving tasks. The simulator can calculate a performance score with a maximum of 100. One study [21], [21] used this simulator.

The last is Doron's AMOS-II, an interactive simulator mainly used for training, e.g., police officers [29]. One of the included studies [22], [22] used it. The simulator provides the overall score that depends on various parameters, e.g., speed and stopping distance. It comes with various pre-built scenarios. The study using it is the only one that did not utilize *errors* as assessment inputs. The reason appears to be that *errors* were not crucial for the original purposes of their study, as their study focused on evaluating impaired self-awareness, where overall scores across aspects potentially already satisfy the requirements of the study, i.e., there is no need to delve further into finer-grained details.

### 3.7 Assessment techniques for driving assessment

An assessment technique transforms the assessment inputs into data that can be used by the evaluation metrics. Technique types found in the review are threshold-based, ML-based, and driving simulator calculation techniques.

#### 3.7.1 Threshold-based techniques

In this type of technique, inputs are computed to be subsequently used by threshold metrics. One study [27], [27] calculated a sum of all driving errors, consisting of collisions, speed exceedances, centerline crossings, road edge excursions, and stop signs missed. Another study [23], [23] also used a sum of errors with six criteria, each criterion having a different maximum penalty score. The penalty score–a lesser score, a better performance–is more interpretable in this sense and allows for a more granular performance classification. The detailed criteria and associated maximum score are shown in Table 3. The last study in this group [13] used an algorithm derived from age-based normative values [30], [31] of all the simulator parameters to derive a performance score.

#### 3.7.2 ML-based techniques

ML-based techniques have harnessed the power of teaching a machine to learn patterns from a set of examples. Such a technique was applied in one study [15]. The study used deep features to extract data and fed them to several classifiers, each in charge of a different performance metric, to capture the holistic driving performance.

#### 3.7.3 Driving simulator calculation techniques

This technique type directly uses a driving simulator that is able to automatically give a performance score after each participant completes the assessment tasks. Score interpretation from a simulator of interest varies depending on the simulator's maker. Doron's AMOS-II, used by McKay et al. [22], [22], and Gridspace's GDS-300 simulator, used by Park [21], [21], are the two simulators in the review where this technique was employed.

### 3.8 Evaluation metrics for driving assessment

The assessment techniques greatly influence associated evaluation metrics. The metrics allow the categorization of the driver's abilities. As a result, it is impossible to know the proprietary metrics for one included study [22], [22] that had the driving simulator directly report the result. In addition, another study [23], [23] did not explicitly specify how they classified the score. Hence, we only report here evaluation metrics for the threshold-based and ML-based techniques.

#### 3.8.1 Threshold-based metrics

In one study [21], [21], a constant value, 80, was defined for the threshold; any score higher than or equal to 80 is PASS; otherwise, FAIL. Two studies [13], [27] used statistical values to define classification ranges. One of them [27], [27] used a threshold of three standard deviations (3*s*) from a mean ($\overset{¯}{X}$), and, in particular, any score above $\overset{¯}{X}+3s$ is FAIL; otherwise, PASS. The other study [13] utilized an algorithm based on normative values to classify PASS and FAIL.

#### 3.8.2 ML-based metrics

One study [15] used ML to help classify the drivers according to the trained models into pre-defined classes. The classification metrics derived from this technique are latent because the classifiers in use are black-box models. The study used 11 binary classifiers to classify 10 driving ability items and 1 driving sustainability item.

## 4 Discussion

In this section, we analyzed the inputs, techniques, and metrics utilized in the studies included in our review. Additionally, we compared our findings with those of the aforementioned existing review and discussed their implications for practical applications. Our review identified several observations that are consistent with previous studies. We also examined the potential of SAAD-PS for policymakers and the advantages it could provide in terms of reducing the cost of driving assessments for post-stroke patients. Finally, we summarized our recommendations as guidelines for reporting and conducting studies in SAAD-PS.

We identified inputs used in SAAD-PS can be categorized into five distinct categories. Several noteworthy observations emerged from our investigation which we discussed in Section 4.3. The analysis of the included studies also revealed the use of three primary assessment techniques: threshold-based, ML-based, and driving simulator calculation techniques, along with two associated metrics, specifically, threshold-based and ML-based metrics. Further discussion on these topics is provided in 4.5.

Nevertheless, limited use of assessment inputs and techniques utilized in the included studies may lead to incomplete evaluations and potentially inaccurate conclusions. We discussed more about this issue in Section 4.7. Another aspect to consider is the inconsistency in assessment inputs utilized across the included studies, with only a few shared inputs being used. The decisions regarding selected inputs therein are additional factors influencing the interpretation of the results, such as whether these inputs are sufficient to draw a conclusive performance grade for each participant. In general, future studies should consider employing a broader range of assessment inputs to gain a more comprehensive understanding of driving abilities and utilize our review as a guideline, discussed more in Section 4.3, to select the most effective inputs for their studies.

Our review and the previous review [9] revealed three significant insights. First, McKay et al.'s study was the only one to be included in both reviews, indicating the scarcity of studies in this area. Second, both reviews highlighted the fact that there were only a limited number of studies in SA, underscoring the pressing need for additional research in these areas.

Third, both the previous review and our study share concerns regarding the validity of utilizing driving simulators for SA and report the practices of articles utilizing SA which lack various details and are inconsistent in aspects that are being reported. We encourage future studies to provide more details when utilizing SA in the aspect of driving simulator and evaluation by following guidelines in this study. We recommend studies report details regarding SAAD-PS following our guideline illustrated in Section 4.7. Comprehensive reporting will not only enhance the reproducibility of the study but also help future studies in analyzing and improving it.

A recent study [17] found that the validity of SA is inconclusive. We conjecture that one reason limiting validity and potentially reliability of the methods is their reproducibility, which is partly due to inconsistency in reporting practices in studies. Therefore, we underscore the importance of reporting these details. We further encourage studies to also publish artifacts, e.g., code, data, and programs utilized for assessment, to further enhance the reproducibility of the study and benefit the community. As the current state of reporting in the literature is inconsistent, we are unable to draw further and more insightful conclusions from the included studies.

### 4.1 General characteristics of included studies

We observed heterogeneity in the purposes of utilizing SAAD-PS across studies. Among the included studies, we can further categorize purposes into three main categories: for comparison among groups, for obtaining ground truth performance, and for obtaining training data. The first category of purpose aimed to utilize SAAD-PS to obtain numerical performance indicators used to establish comparisons among groups of participants, such as between post-stroke and healthy controls or between subtypes of stroke. Similarly, the second category also aimed to obtain numerical performance indicators, but this time the performance is utilized to find associations with other metrics or tests, including cognitive tests. Finally, the last category aimed to utilize obtained performance as a target for training models, e.g., ML models. Differences in purposes also reflect in differences in decisions made regarding simulator tools, assessment inputs, driving scenarios, assessment techniques, and evaluation criteria.

In particular, we found that a higher number of inputs were collected in a study by Jeon et al. [15] which is to be expected as ML models, specifically, neural networks, have high data requirements [32], and there is less need to concern about manual feature engineering as ML can automatize this aspect. On the other hand, the rest of the studies focused more on choosing assessment inputs that will more accurately reflect the performance of participants in their objectives, for example, focusing on the number of errors as in a study by Park [21], [21].

The influence of objectives also reflects in the design of driving scenarios. We observed most studies utilized more than one area and driving scenarios. We argue that this is due to reducing bias and to obtain a more generalized performance that can reflect real-world settings which contain a greater variety of scenarios. This also reflects the strength of driving simulators in allowing any amount and designs of scenarios. As for a study by Jeon et al. [15] which did not directly aim to assess participants' performance, a higher variety of scenarios attributed to a greater variety of data patterns which leads to better generalization of the trained model, i.e., less chances of overfitting.

On another aspect, we observed all included studies had participants in the age range of middle age to elderly. This age range limits implications made throughout the study in the younger population when utilizing SAAD-PS. We prompt researchers to conduct further studies in a younger group of the population than current state as the World Stroke Organization previously reported [33] that “each year, over 16% of all strokes occurs in people 15–49 years of age,” which is a non-negligible amount of the population to be considered. Additionally, driving ability is directly linked to freedom and independence [4], which may be more crucial in this younger population, as they have years ahead to live with these potentially restricted capabilities.

### 4.2 Driving scenarios

We found that the included studies did not provide many details on the rationales behind the designs of driving scenarios utilized in their studies. We also found that the included studies utilized a variety of driving scenarios which we conjectured that the studies tried to comprehensively assess their participants, as can be observed from a trend of diverse areas and tasks utilized to assess participants. Another factor contributing to these inconsistencies in implementing driving scenarios across studies is the fact that each area (country, state, etc.) has different requirements expected from a driver as compared to the driving licensing exam. While OA is considered a gold standard [11], it is obvious that a driving license exam is a required process in order to drive legally. Nevertheless, different countries implement driving license exams differently and could lead to bias in implementing driving scenarios across studies, aside from differences in objectives of each study.

We also found a consistent observation with a previous review on driving scenarios implemented for SA [6] that *urban* is the most common driving area implemented, as it is an area implemented by all of our included studies. As stated in the previous review [6], *urban* driving areas are usually coupled with denser traffic, providing more challenging scenarios for participants. This may be because urban areas tend to share similar features regardless of countries and regions, and are commonly available in driving simulators. Nevertheless, it is important to note that while driving simulators provide more flexibility in implementing driving scenarios compared to OA, which requires actual establishments, there is a limit posed by the driving simulator on what kind of driving scenarios could be implemented. This is especially true for commercial driving simulators as utilized by all included studies. As pointed out by the existing review [6], “the driving scenarios and environmental settings that can be introduced in the assessment may not be varied due to the limit in the configuration of the driving simulator.”

### 4.3 Assessment inputs

Similar to driving scenarios, assessment inputs are varied by objectives of the study and a driving simulator utilized in each study. Furthermore, assessment inputs were also similar to driving scenarios in the sense that they influence interpretations of outcomes. Therefore, inconsistency hinders meta-analysis of multiple studies. Nevertheless, we observed *error* as the most popular choices across the included studies. We hypothesize that this preference for *error* may stem from its alignment with real-world assessments, such as those commonly encountered in driving licensing exams, as well as its reflection of behaviors prohibited by regulations and laws.

For example, centerline crossings in normal circumstances are considered illegal, e.g., Section 43 of Thailand's Land Traffic Act BE 2565^2^; exceeding speed limits is also prohibited, e.g., Article 22 of Japan's Road Traffic Act 1960^3^; and failing to use a seat belt results in fines in the United States.^4^ These examples underscore the significance of inputs categorized as *error* in assessing the fitness of post-stroke patients for driving. In fact, these inputs may be the most crucial factors. Currently, there is no standard for determining which inputs should be collected when designing SAAD-PS. However, we recommend that practitioners and researchers consider collecting inputs related to the “Error” category by designing SAAD-PS in accordance with relevant local or national laws and regulations. Similar to OA, which integrates multiple driving scenarios and measures various aspects resembling part of a driving licensing exam (e.g., [34], [35], [36]), we believe that SA should also follow this approach. By doing so, we anticipate that the validity of SA could be further enhanced.

Furthermore, SA has advantages over OA in implementing more challenging driving scenarios [9] and collecting metrics that cannot be easily collected in OA due to limitations in physical space, equipment, and safety concerns. This aspect is particularly important because ML-based techniques often rely on adequate amounts of data. However, inconsistency and lack of data disclosure in studies also impede the development of ML-based techniques. In other related fields such as robotics [37], medical imaging [38], and computer vision [39], we observe various efforts in open data aggregation to support further development of ML-based approaches. As ML-based approaches hold various benefits over traditional analysis and Jeon et al. [15] have already taken the first step towards this line of research, we encourage future studies to open-source their code and raw data to further support this effort.

### 4.4 Simulator tools

We observed that all included studies utilized commercial driving simulators, with STISIM Drive® being the most popular choice among them. We conjecture that those studies utilized this simulator due to its popularity and ease of setup. As previously discussed, the primary aim was to obtain ground truth or performance indicators for various purposes. Therefore, there is less emphasis on meticulously choosing the simulator.

Furthermore, with a lack of standards in designing driving scenarios and selecting assessment inputs, researchers often rely on what the simulators can offer, which is generally sufficient for the majority of cases with these types of objectives. However, we believe that emphasis should be placed on selecting the appropriate simulator based on the study's specific needs. Nevertheless, this cannot be confirmed as the studies often lack reporting deeper rationales behind choosing a particular type of simulator.

We also want to point out that there are various studies currently exploring open-source and/or low-cost driving simulators [40], [41], [42]. These types of simulators offer higher flexibility in designing driving scenarios and choosing inputs to be collected. While these types of simulator also come with the cost of higher overhead in setup, it lowers the barrier to implementing such a system by utilizing various cheaper equipment and flexibility in designing the suite of hardware to match the needs.

For example, researchers have flexibility in choosing the output screen, such as how many monitors they want and what the setup of those monitors will be, or instead opting for a projector screen. They can also choose to incorporate modified pedals to offer a more realistic experience for participants who require special modifications of a vehicle. Furthermore, it is possible for researchers to utilize a laptop to power the simulation session due to the low-cost nature of these types of simulators and also to gain higher portability.

Furthermore, utilizing this kind of simulator also lowers the cost of maintenance as components can be found in general markets instead of relying on custom builds from specific brands. Additionally, utilizing open-source simulators allows researchers more ease in open-sourcing their code and data. By open-sourcing these artifacts, it is easier for future studies to reuse scenarios and sets of assessment inputs, further supporting the goal of establishing a common platform and guidelines for this line of research.

### 4.5 Assessment techniques and evaluation metrics

We classified assessment techniques and associated evaluation metrics into three main categories: threshold-based, ML-based, and driving simulator calculation approaches. We found that the threshold-based and driving simulator calculation approaches are utilized by all included studies that did not focus on developing a model for driving ability classification. Both approaches offer simplicity in implementation, which is adequate for the objectives of these studies. Driving simulators were utilized as tools for assessing performance and were not the main focus, unlike the study by Jeon et al. [15]. Their study focused more on developing novel models used for classifying various ability metrics and fitness to drive, leading to the use of more advanced techniques like ML, which helps in processing larger amounts of data. Nevertheless, we found that all approaches enable researchers to achieve the same goal of assessing the ability of participants and classifying a grade for them as PASS or FAIL.

To assist future studies in choosing an appropriate approach for their own research, we summarize the strengths and considerations of each approach in Table 5. In general, the choice of approach depends on two main factors: flexibility and the amount of available input data. First, researchers should assess the level of flexibility required to satisfy their objectives. This involves determining whether the driving scenarios and assessment inputs collected are commonly available in commercial simulators or not.

**Table 5:** Strengths and considerations of each assessment technique. **T** denotes threshold-based technique, **ML** denotes ML-based technique, and **DSC** denotes driving simulator calculation.

	Strengths	Considerations
T	**Table:** + Easy to adjust thresholds + Easy to interpret results + Straightforward logic	**Table:** - Challenging in selecting threshold values to use - Inputs often require manual pre-/post-processing

ML	**Table:** + More manageable in handle large amount of inputs + Able to work with latent patterns	**Table:** - Some ML approaches are data hungry (training) - Some ML approaches are hard to interpret results (black box)

DSC	**Table:** + Minimal overhead in setup + Commercial support	**Table:** - Reduced flexibility in configurations - Limited access to implementation details

Additionally, researchers should consider whether the calculation approach is suitable for their study or not, as some details may not be disclosed, which hindered the interpretation of the results. If a study can be conducted using commonly available scenarios and inputs found in commercial driving simulators, and the focus is solely on the final grade of PASS or FAIL, choosing the driving simulator calculation approach would yield the least overhead and the most simplicity, albeit at the monetary cost of the driving simulator hardware and/or software, as well as relinquishing some flexibility. However, if more flexibility in manipulating metrics or analysis beyond those typically offered by commercial driving simulators is required, implementing a calculation approach using either threshold-based or ML-based methods may be more suitable. This leads to the second factor: the amount of available data. Given the scarcity of ML models in this field at the current state, and the absence of available pre-trained models, the amount of quality data becomes a critical factor in determining the success of an ML-based approach. ML models require a sufficient amount of data to train a decent model. Nonetheless, the threshold-based approach may be most suitable for most types of research in this field at present.

It is crucial to note that the threshold-based approach should always perform an ablation study or have a strong rationale when choosing a threshold value, as previously stated. This is crucial for reproducibility and interpretation of the results. Similarly, ML-based techniques, which suffer from interpretation issues due to its black-box nature, should also consider incorporating explainable ML techniques [43]. Furthermore, we would like to restate that techniques themselves are also limited by the choice of driving simulator. Thus, decisions may not always be straightforward.

### 4.6 Broader implications

We would like to acknowledge that in the current state, no approaches to driving assessment are perfect for post-stroke drivers. Post-stroke patients suffer from various degradations in their capabilities, including their physical, mental, and perceptual capabilities [44], [45], [46], [47]. All of these have been partially assessed in independent settings through various types of driving assessments, which is reflected in some studies that observed an interesting result: the performance obtained from one type of driving assessment may not accurately predict performance in another type of assessment [17], [34]. In particular, a study [34] found that post-stroke patients who performed poorly in a cognitive assessment may perform decently in an on-road assessment, which is more crucial as it directly reflects their actual driving skills. However, in our opinion, we believe that the obtained cognitive assessment results are also non-negligible as they can pose some risks associated with driving.

Driving is a complex activity and requires a multitude of skills. Impaired cognitive abilities post-stroke may hinder some abilities to resume driving [48], which CA, SA, or OA alone may not be able to detect certain aspects due to the lack of, or limited correlation between, each assessment approach [17], [49], [50]. These SA and OA only assess capabilities at the activity levels of the International Classification of Functioning, Disability, and Health (ICF) [51], which may not be enough. We argue that instead of utilizing CA and SA as a pre-screening tool, e.g., [15], [28], it may be more beneficial to assess with all available approaches coupled with an analysis of body checkup reports, as each approach assesses different aspects of post-stroke patients, including those falling under the ICF's body structures and functions, which may not be able to be circumvented through rehabilitation or training as straightforwardly as driving skills.

With the increased amount of data obtained from tests, ML models may be aided in data analysis and provide initial recommendations, specifically in low-risk tests such as CA and SA. Furthermore, we note the potential of utilizing large language models, as they have demonstrated competencies [52], [53], [54], and applications [55] in medical domains, along with their limitations, including hallucinations and knowledge gaps [56]. Therefore, these models are only suitable for low-risk environments, and expert supervision is required. In fact, this consideration applies to ML models in medical applications in general, owing to various ethical considerations [57]. Nevertheless, integrating these models into processes potentially reduces the cost of operations and speeds up or automates various processes, ultimately making assessment more accessible to all.

Despite its limitations, SAAD-PS offers policymakers new opportunities, particularly in the context of post-stroke driving regulations. Currently, many countries lack clear guidelines that mandate post-stroke patients to reassess their driving abilities after an incident. A country with regulations like Australia [58] requires post-stroke drivers to obtain clearance from medical personnel, which often imposes a high workload on them in their day-to-day activities [59], instead of relying solely on standard driving assessments or exams.

Nevertheless, the driving exam is a process associated with high costs for both test-takers, staff, and policymakers. This cost includes operating costs for on-road driving assessment facilities and examination fees, which are often burdensome for test takers. Facility operating costs include expenses such as staff stipends, equipment maintenance fees, and infrastructure fees, and these costs are often passed on to patients in the form of high examination fees. Given that post-stroke patients already face significant financial burdens [60] for recovery as well as rehabilitation programs and equipment, the additional cost of a driving examination can be a significant burden. However, SAAD-PS has the potential to mitigate this issue by reducing the cost of driving exam facility operations, which would in turn decrease the examination fee for driving tests.

Another factor that could contribute to the lack of regulation implementation is the adaptation of the test itself to the unique aspects of post-stroke drivers. As previously mentioned, post-stroke patients suffer from various impairments. In many cases, they require special supportive equipment or modifications to a vehicle. While it is possible to modify one's own vehicle, this modification may pose a challenging concern, as modifications to the equipment used for standardized tests for post-stroke drivers may be treated as illegal in some cases or simply lack understanding or knowledgeable staff in bringing these special requirements to life.

Another aspect to consider is that while it may be possible to retrain post-stroke patients to regain their driving abilities, physical and cognitive changes inflicted by stroke itself may not be fully recoverable through treatment and rehabilitation. Governments should take this into account and may require certain types of impaired drivers to follow specific regulations, such as using specifically designed pedals or knobs instead of regular ones to assist in driving. Furthermore, we propose that tests implemented and required by laws or regulations to assess fitness to drive for post-stroke drivers should be able to report performance on a spectrum instead of binary outcomes, i.e., PASS or FAIL. This way, it is possible to adapt the requirements after testing in cases where some post-stroke drivers may require additional or modified equipment to be able to get back on the road while ensuring the safety of all road users and maintaining a higher level of freedom associated with the quality of life for post-stroke drivers [61].

### 4.7 Guidelines

In this section, we summarize our recommendations made throughout this study for future studies. We separate our recommendations for two different purposes: (1) studies interested in utilizing SAAD-PS in the future and (2) future studies aimed at addressing current gaps in SAAD-PS related research. These two aspects are detailed in Sections 4.7.1 and 4.7.2, respectively.

#### 4.7.1 Utilizing SAAD-PS

We offer guidelines on what should be reported for studies utilizing SAAD-PS. We provide details for different aspects that some studies may choose to delve into, focusing only on what is applicable to them. However, we believe that for the majority of studies, they should consider all aspects when reporting.

1. **Driving scenarios**: studies should consult an existing review [6] on what items they should report, such as driving area, weather, and traffic conditions. Furthermore, they should report on the study process from start to finish and its applicability, i.e., when to use each scenario. Rationales for the design and decisions of each scenario should be included. Additionally, the approximate time spent and course length for each scenario should also be reported so that readers of these studies have a better understanding and interpretation of the results.
  In this review, we also found another consistency regarding practice sessions. We believe that in the majority of cases, practice sessions should be included, and details of these sessions should be provided. Furthermore, we argue that practice sessions should not be the ones used for actual testing sessions in the majority of studies, although there might be exceptions in some cases which should include the rationale for making such decisions.
2. **Assessment inputs**: each assessment input will affect the choice of assessment techniques and evaluation metrics, as well as how readers can interpret the applicability of the study. Therefore, researchers should report on what those inputs are, how they collect such inputs (e.g., expert observation, sensor monitoring, or machine calculation), and the rationales behind the decision to include or not include such inputs, as well as the reasons for designing the input in such a way. The range of values and data types of the inputs should also be included in the report.
3. **Simulator tools**: the choice of a driving simulator is crucial, as sometimes the simulator can affect various aspects such as available driving scenarios, assessment inputs, and assessment techniques. Ultimately, some simulators may not support all decisions dictated by the objectives of the study. Therefore, once the decision on the simulator has been made, details and rationales should be included.
  This includes, but is not limited to, rationales for choosing the simulator, setup details (e.g., brand, model, software and hardware version, number of monitors, screen size, pedals, steering wheels, positioning of the simulator, inputs and outputs of the simulator, and conditions of the place for stationing the simulator). If possible, an image of the setup should be included in the study. Furthermore, if additional hardware is added or modifications are made to the simulator, they should be noted, and rational support for such decisions should be provided in the report.
4. **Assessment techniques and evaluation metrics**: similar to all other aspects, decisions made for the techniques and metrics greatly affect the interpretation of outcomes in a study. We argue that even in studies where the assessment process is not the main focus and simply utilizes SAAD-PS to obtain performance indicators, it is still relevant to provide such details. This includes how assessment inputs are processed as dictated by the technique. Pre-processing applied to the inputs before utilizing such techniques or post-processing applied to the outcomes before judging by the metrics should be included. Possible class outputs from the metrics and how to interpret them should also be included.
  For studies utilizing a threshold-based approach, rationales for choosing the cut-off values (either through ablation studies, pilot experiments, or supported evidence in existing studies) should be reported. For studies utilizing an ML-based approach, model training details such as hyperparameters, dataset, and evaluation paradigm should be detailed. Consult the model card [62] for what information should be included. Finally, for studies utilizing a driving simulator calculation approach, they should report on specific features of the simulator utilized to obtain the performance results and, if applicable, how it derives such results from the inputs.

#### 4.7.2 Addressing gaps in SAAD-PS

In contrast to the previous section, in this section, we offer guidelines for addressing gaps in SAAD-PS identified through our review that future studies should consider. These include general gaps as well as those specific to certain aspects of SAAD-PS. We hope that by listing these gaps here, they will be overcome over time with increased interest in this line of research.

• Research in this SAAD-PS domain is scarce. Even SA in general lags behind other approaches, CA and OA. This leads to two cascading issues down the line. First, the limited settings of SAAD-PS in studies also restrict their validity and applicability, which can be overcome with more studies in broader settings. Second, there is limited research data available. In an era of ML powered by enormous amounts of data to develop useful models, the limited amount of data or lack of disclosure of such data hinders the development of such approaches. However, it is crucial to note that when data is opened, all sensitive information should be eliminated or obfuscated.
• Related to the first point, the practice in reporting details relevant to the assessment process is subpar. This limits understanding of readers and reduces the reproducibility and reusability of such research. For further details, please consult our guidelines in the previous section.
• As previously discussed, while strokes may be prominent in the older population, they also occur in younger individuals. Based on the included studies, we emphasize that there is a lack of research utilizing SAAD-PS in the younger population. Since driving ability may be even more crucial in younger individuals, researchers should also invest effort in understanding this group.
• As previously discussed, each type of assessment can only capture certain aspects of the abilities relevant to driving. Driving is multifaceted and requires various skills to be sufficiently able to drive safely on the road. We recommend practitioners to comprehensively utilize all information from tests and reports of a certain patient in determining whether they can safely return to the road and not solely rely on a single type of test.
• Policymakers should consider SAAD-PS as part of the tools that can be utilized in assessing the driving abilities of post-stroke drivers. Laws or regulations should be made mandatory to ensure the safety of all road users, including post-stroke drivers. However, we acknowledge that practices may vary by country.
• **Driving scenarios**: studies should explore further on guideline for designing driving scenarios, e.g., should be it designed in similar spirit to a driving licensing exam or should be designed specifically to assess certain aspects unique to post-stroke drivers.
• **Assessment inputs**: while it seems that the majority of the included studies utilized inputs of the “Error” category, the validity of such inputs cannot be conclusively determined. Therefore, future studies should also investigate the correlation between each assessment input and its impact on overall performance or the interpretation of the study. Similar to driving scenarios, assessment inputs may also be designed similarly to a driving licensing exam, although their validity needs to be further investigated.
• **Simulator tools**: while all included studies utilized commercial driving simulators, we believe that low-cost or open-source driving simulators may also offer adequate or even better alternatives. Open-source simulators usually provide more flexibility in designing driving scenarios and collecting assessment inputs. Although there are currently some aspects in which open-source simulators lag behind commercial ones, continuous efforts are being made to close this gap. Therefore, we recommend that future studies consider this alternative.
• **Assessment techniques and evaluation metrics**: currently, all included studies reported performance indicators as binary outcomes, i.e., either PASS or FAIL. This approach may have limited benefits. We agree that a clear-cut outcome is crucial in classifying whether such a person undertaking the assessment is suitable to go back on the road or not. However, this approach may be limited in rehabilitation or training scenarios.
  Binary outcomes can only indicate if a certain person is adept enough to drive or not, but not how they can improve if they fail. We see some efforts [15] in trying to overcome this issue by having multiple classifiers for each driving aspect. While this approach has its own merits, we propose another alternative solution by designing evaluation metrics to output a spectrum, i.e., a range of possible classes. This spectrum may be easily achieved by dividing driving scores using certain thresholds or simply reporting scores with a range of possible values in addition to the binary class. This way, both test-takers and proctors can assess their development and identify points to be improved.

### 4.8 Limitations

Information about post-stroke patients is rather limited. This is because not all of the included studies provided the exact details on the type of stroke and the duration after stroke; these two aspects are crucial to associate metrics used across all the included studies and how effective those metrics have on specific populations. This review has another limitation due to the “English-only” search criterion and the “journal article” search parameter, which might result in a small number of the included studies.

## 5 Conclusion

After reviewing six articles that examined the input data, techniques, and metrics for SAAD-PS, our analysis highlighted that most of the included studies analyzed the post-stroke patients' performance, usually, with healthy controls. Only one study focused on an automatic assessment system. Our findings revealed the necessity for future research to focus on various aspects of SAAD-PS, including the reporting of the assessment process's details, the validity of the system, and an overall study of SAAD-PS. From a practical perspective, addressing these research gaps could enable the integration of SAAD-PS into driving exam centers, thereby reducing operational costs and increasing the chances of regulated post-stroke driving, contributing to safer roads.

## Funding

This work was supported in part by the 10.13039/501100005405Ritsumeikan University's Research Advancement Research Promotion Program for Acquiring Grants-in-Aid for Scientific Research.

## Declaration of generative AI and AI-assisted technologies in the writing process

During the preparation of this work the authors used ChatGPT^5^ in order to address grammatical issues and brainstorm writing ideas. After using this service, the authors reviewed and edited the content as needed and take full responsibility for the content of the publication.

## CRediT authorship contribution statement

**Pittawat Taveekitworachai:** Writing – original draft, Resources, Methodology, Investigation, Data curation, Conceptualization. **Gunt Chanmas:** Resources, Methodology, Investigation, Conceptualization. **Pujana Paliyawan:** Writing – review & editing, Validation, Methodology, Conceptualization. **Ramita Thawonmas:** Writing – review & editing, Validation. **Chakarida Nukoolkit:** Writing – review & editing, Validation, Project administration, Methodology, Conceptualization. **Piyapat Dajpratham:** Writing – review & editing, Validation, Methodology, Conceptualization. **Ruck Thawonmas:** Writing – review & editing, Validation, Supervision, Project administration, Methodology, Funding acquisition, Conceptualization.

## Declaration of Competing Interest

The authors declare the following financial interests/personal relationships which may be considered as potential competing interests:

Ruck Thawonmas reports financial support was provided by 10.13039/501100005405Ritsumeikan University. Ruck Thawonmas also reports that he serves as an Associate Editor for the Computer Science Section of Heliyon. If there are other authors, they declare that they have no known competing financial interests or personal relationships that could have appeared to influence the work reported in this paper.

## Data Availability

Data are included in this article and its supplementary materials.

## Appendix A Supplementary material

MMC Search terms and search parameters. MMC

## Footnotes

1 https://rayyan.ai.

2 https://www.ratchakitcha.soc.go.th/DATA/PDF/2565/A/028/T_0005.PDF.

3 https://www.japaneselawtranslation.go.jp/en/laws/view/2962/en#je_ch3sc2at1.

4 https://www.iihs.org/topics/seat-belts/seat-belt-law-table.

5 http://chat.openai.com.
