# Supplementary material for: A systematic review of major evaluation metrics for simulator-based automatic assessment of driving after stroke
Source: Heliyon. 2024 Jun 17;10(12):e32930. doi: 10.1016/j.heliyon.2024.e32930 (PMC11252877; doi:10.1016/j.heliyon.2024.e32930)
Supplement: MMC — Search terms and search parameters. [file mmc1.docx]

**Table S1** Search terms and search parameters

| **Database** | **Search terms** | **Parameters** |
| --- | --- | --- |
| PubMed | ([stroke](mailto:https://www.ncbi.nlm.nih.gov/mesh/68020521) OR cerebrovascular OR "cerebral vascular") AND (driving OR drive) AND simulat* AND (test OR assess* OR measur* OR evaluat*) | Text availability: Full text Article type: Research support, review, systematic review Specie: Human Language: English Journal: MEDLINE  Result by year: 2010 - 2021  Result by year for updated search: 2022 or 2023 |
| Web of Science | (((ALL=(stroke OR cerebrovascular OR "cerebral vascular")) AND ALL=(driving OR drive)) AND ALL=(simulat*)) AND ALL=(test OR assess* OR measur* OR evaluat*) | Document type: Articles, review articles Language: English  Timespan: 2010 – 2021  Timespan for updated search: 2022 or 2023 |

**Table S1** Search terms and search parameters

| **Database** | **Search terms** | **Parameters** |
| --- | --- | --- |
| ScienceDirect | drive AND (simulator OR simulate) AND (test OR assess OR measure OR evaluate) | Three separate search due to limitation on number of operators:  1. Title, abstract or author-specified keywords: stroke + *main search terms*  2. Title, abstract or author-specified keywords: cerebrovascular + *main search terms*  3. Title, abstract or author-specified keywords: cerebral vascular + *main search terms*  Year(s): 2010 – 2021  Year(s) for updated search: 2022 or 2023 |

**Table S1** Search terms and search parameters

| **Database** | **Search terms** | **Parameters** |
| --- | --- | --- |
| ACM Digital Library | [[All: stroke] OR [All: cerebrovascular] OR [All: "cerebral vascular"]] AND [[All: driving] OR [All: drive]] AND [All: simulat*] AND [[All: test] OR [All: assess*] OR [All: measur*] OR [All: evaluat*]] AND [Publication Date: (01/01/2010 TO 12/31/2021)] | Journals Content type: Research articles  In the first updated search, change “AND [Publication Date: (01/01/2010 TO 12/31/2021)]” to “AND [Publication Date: (01/01/2022 TO 06/21/2022)]”  In the second updated search, change “AND [Publication Date: (01/01/2010 TO 12/31/2021)]” to “AND [Publication Date: (22/06/2022 TO 31/10/2022)]”  In the third updated search, change “AND [Publication Date: (01/01/2010 TO 12/31/2021)]” to “AND [Publication Date: (1/11/2022 TO 31/12/2022)]”  In the fourth updated search, change “AND [Publication Date: (01/01/2010 TO 12/31/2021)]” to “AND [Publication Date: (01/01/2023 TO 31/12/2023)]” |
| IEEE Xplore Digital Library | (stroke OR cerebrovascular OR "cerebral vascular") AND (driving OR drive) AND simulat* AND (test OR assess* OR measur* OR evaluat*) | Journals  Year: Range: 2010 – 2021  Year for updated search: Single Year: 2022 or 2023 |

**Table S2** Data extraction template

| **Reference** | **Objectives** | **Simulator Tool** | **Assessment Inputs** | **Driving Scenarios** | **Assessment Techniques** | **Evaluation Criteria** | **Results** | **Participant Demographic** |
| --- | --- | --- | --- | --- | --- | --- | --- | --- |
|  |  |  |  |  |  |  |  |  |
|  |  |  |  |  |  |  |  |  |
|  |  |  |  |  |  |  |  |  |
|  |  |  |  |  |  |  |  |  |
|  |  |  |  |  |  |  |  |  |

**Table S3** Assessment inputs used in the study

**Type of Inputs**: E = Error, T = Time, S = Speed and Acceleration, V = Vehicle Properties, and C = Count. In each cell, "O" denotes the presence of such input in the study, and "X" denotes the absence of such input.

| **Inputs/Study** | **Hird et al. (2018)** | **Jeon et al. (2021)** | **Motta et al. (2014)** | **McKay et al. (2011)** | **Akinwuntan et al. (2013)** | **Park (2015)** |
| --- | --- | --- | --- | --- | --- | --- |
| Centerline crossings  (n = 4) (E) | O | X | O | X | O | O |
| Road edge excursions  (n = 3) (E) | O | X | O | X | O | X |
| Speed exceedances  (n = 3) (E) | O | X | X | X | O | O |
| Collisions  (n = 2) (E) | O | X | X | X | O | X |
| Brake reaction  (n = 2) (T) | X | X | X | X | X | O |

**Table S3** Assessment inputs used in the study

| **Inputs/Study** | **Hird et al. (2018)** | **Jeon et al. (2021)** | **Motta et al. (2014)** | **McKay et al. (2011)** | **Akinwuntan et al. (2013)** | **Park (2015)** |
| --- | --- | --- | --- | --- | --- | --- |
| Number of  collisions with  pedestrians  (n = 2) (E) | X | X | O | X | O | X |
| Stop signs  missed  (n = 1) (E) | O | X | X | X | X | X |
| Vehicle  obstruction  (n = 1) (E) | X | X | X | X | X | X |
| Pedestrian  obstruction  (n = 1) (E) | X | X | X | X | X | X |
| Driver’s  longitudinal  acceleration  (n = 1) (S) | X | O | X | X | X | X |

**Table S3** Assessment inputs used in the study

| **Inputs/Study** | **Hird et al. (2018)** | **Jeon et al. (2021)** | **Motta et al. (2014)** | **McKay et al. (2011)** | **Akinwuntan et al. (2013)** | **Park (2015)** |
| --- | --- | --- | --- | --- | --- | --- |
| Driver’s lateral  acceleration  (n = 1) (S) | X | O | X | X | X | X |
| Driver’s  longitudinal  velocity (ft/s)  (n = 1) (S) | X | O | X | X | X | X |
| Driver’s lateral  velocity (n = 1) (S) | X | O | X | X | X | X |
| Driver’s lateral  lane position  with respect to the roadway  dividing line  (n = 1) (V) | X | O | X | X | X | X |

**Table S3** Assessment inputs used in the study

| **Inputs/Study** | **Hird et al. (2018)** | **Jeon et al. (2021)** | **Motta et al. (2014)** | **McKay et al. (2011)** | **Akinwuntan et al. (2013)** | **Park (2015)** |
| --- | --- | --- | --- | --- | --- | --- |
| Vehicle  curvature  (n = 1) (V) | X | O | X | X | X | X |
| Current  roadway  curvature  (n = 1) (V) | X | O | X | X | X | X |
| Vehicle heading angle  (n = 1) (V) | X | O | X | X | X | X |
| Steering wheel  angle input  (n = 1) (V) | X | O | X | X | X | X |

**Table S3** Assessment inputs used in the study

| **Inputs/Study** | **Hird et al. (2018)** | **Jeon et al. (2021)** | **Motta et al. (2014)** | **McKay et al. (2011)** | **Akinwuntan et al. (2013)** | **Park (2015)** |
| --- | --- | --- | --- | --- | --- | --- |
| Longitudinal  acceleration due to the throttle  (n = 1) (S) | X | O | X | X | X | X |
| Longitudinal acceleration due to the brakes  (n = 1) (S) | X | O | X | X | X | X |
| Running  compilation of the crashes that the driver has been involved in  (n = 1) (E) | X | O | X | X | X | X |

**Table S3** Assessment inputs used in the study

| **Inputs/Study** | **Hird et al. (2018)** | **Jeon et al. (2021)** | **Motta et al. (2014)** | **McKay et al. (2011)** | **Akinwuntan et al. (2013)** | **Park (2015)** |
| --- | --- | --- | --- | --- | --- | --- |
| Driver’s  longitudinal  velocity (mph)  (n = 1) (S) | X | O | X | X | X | X |
| Steering input  counts (Actual  raw input)  (n = 1) (C) | X | O | X | X | X | X |
| Throttle input  counts (Actual  raw input)  (n = 1) (C) | X | O | X | X | X | X |
| Braking input  counts (Actual  raw input)  (n = 1) (C) | X | O | X | X | X | X |

**Table S3** Assessment inputs used in the study

| **Inputs/Study** | **Hird et al. (2018)** | **Jeon et al. (2021)** | **Motta et al. (2014)** | **McKay et al. (2011)** | **Akinwuntan et al. (2013)** | **Park (2015)** |
| --- | --- | --- | --- | --- | --- | --- |
| Steering wheel  rate (n = 1) (V) | X | O | X | X | X | X |
| Minimum time to collision  between the  driver and all  vehicles in the  driver’s  direction  (n = 1) (T) | X | O | X | X | X | X |
| Minimum range  between the  driver and all  vehicles in the  driver’s  direction  (n = 1) (V) | X | O | X | X | X | X |

**Table S3** Assessment inputs used in the study

| **Inputs/Study** | **Hird et al. (2018)** | **Jeon et al. (2021)** | **Motta et al. (2014)** | **McKay et al. (2011)** | **Akinwuntan et al. (2013)** | **Park (2015)** |
| --- | --- | --- | --- | --- | --- | --- |
| Minimum time  to collision  between the  driver and all  vehicles  opposing the  driver’s  direction  (n = 1) (T) | X | O | X | X | X | X |
| Minimum range  between the  driver and all  vehicles  opposing the  driver’s  direction  (n = 1) (V) | X | O | X | X | X | X |

**Table S3** Assessment inputs used in the study

| **Inputs/Study** | **Hird et al. (2018)** | **Jeon et al. (2021)** | **Motta et al. (2014)** | **McKay et al. (2011)** | **Akinwuntan et al. (2013)** | **Park (2015)** |
| --- | --- | --- | --- | --- | --- | --- |
| Number of  collisions,  excluding  pedestrians  (n = 1) (E) | X | X | O | X | X | X |
| Driver stops at  an appropriate  distance from  traffic lights,  stop signs and  obstacles  (n = 1) (C) | X | X | O | X | X | X |

**Table S3** Assessment inputs used in the study

| **Inputs/Study** | **Hird et al. (2018)** | **Jeon et al. (2021)** | **Motta et al. (2014)** | **McKay et al. (2011)** | **Akinwuntan et al. (2013)** | **Park (2015)** |
| --- | --- | --- | --- | --- | --- | --- |
| Driver did not  overtake when unsafe allowed adequate room, and stopped at yellow traffic lights  (n = 1) (C) | X | X | O | X | X | X |
| Kilometers over speed limit  (n = 1) (E) | X | X | O | X | X | X |

**Table S3** Assessment inputs used in the study

| **Inputs/Study** | **Hird et al. (2018)** | **Jeon et al. (2021)** | **Motta et al. (2014)** | **McKay et al. (2011)** | **Akinwuntan et al. (2013)** | **Park (2015)** |
| --- | --- | --- | --- | --- | --- | --- |
| Driver  appropriately  used indicators  to give warning  and future  diverging  movements  (n = 1) (C) | X | X | O | X | X | X |
| Speed  (n = 1) (S) | X | X | X | O | X | X |
| Stop distance  (n = 1) (V) | X | X | X | O | X | X |

**Table S3** Assessment inputs used in the study

| **Inputs/Study** | **Hird et al. (2018)** | **Jeon et al. (2021)** | **Motta et al. (2014)** | **McKay et al. (2011)** | **Akinwuntan et al. (2013)** | **Park (2015)** |
| --- | --- | --- | --- | --- | --- | --- |
| Lane placement  (n = 1) (V) | X | X | X | O | X | X |
| Traffic signal  use (n = 1) (C) | X | X | X | O | X | X |
| Hazard  avoidance  (n = 1) (C) | X | X | X | O | X | X |
| Obeying traffic  signs and  signals  (n = 1) (C) | X | X | X | O | X | X |
| Time to  collision  (n = 1) (T) | X | X | X | X | O | X |

**Table S3** Assessment inputs used in the study

| **Inputs/Study** | **Hird et al. (2018)** | **Jeon et al. (2021)** | **Motta et al. (2014)** | **McKay et al. (2011)** | **Akinwuntan et al. (2013)** | **Park (2015)** |
| --- | --- | --- | --- | --- | --- | --- |
| Total runtime  (n = 1) (T) | X | X | X | X | O | X |
| Simple reaction time  (n = 1) (T) | X | X | X | X | O | X |
| Complex  reaction time  (n = 1) (T) | X | X | X | X | O | X |
| Failed to use  seat belt  (n = 1) (E) | X | X | X | X | X | O |
| Turn signal  errors  (n = 1) (E) | X | X | X | X | X | O |

**Table S3** Assessment inputs used in the study

| **Inputs/Study** | **Hird et al. (2018)** | **Jeon et al. (2021)** | **Motta et al. (2014)** | **McKay et al. (2011)** | **Akinwuntan et al. (2013)** | **Park (2015)** |
| --- | --- | --- | --- | --- | --- | --- |
| Drop out the  course  (n = 1) (E) | X | X | X | X | X | O |
| Accidents  (n = 1) (E) | X | X | X | X | X | O |
